# SPINK-1 Polymorphism as a Pancreatitis Risk Factor

**DOI:** 10.7759/cureus.3852

**Published:** 2019-01-08

**Authors:** Leon D Averbukh, Marianna G Mavilia

**Affiliations:** 1 Internal Medicine, University of Connecticut Health Center, Hartford, USA

**Keywords:** spink-1, pancreatitis, genetics

## Abstract

Pancreatitis in both acute and chronic variants is a common health concern in the US as well as globally. While the most common etiologies for disease remain gallstone impaction in the common bile duct and alcohol abuse, recent studies have shown that genetics may play a significant role as well. Unfortunately, this correlation is not clearly defined and at present, we lack the ability to identify which patients with known pancreatic genetic polymorphisms will develop pancreatitis. We describe the case of a middle-aged male who presented with recurrent pancreatitis in the setting of the serine peptidase inhibitor, Kazal type 1 (SPINK-1) genetic polymorphism. Additionally, we discuss the mechanism behind SPINK-1 polymorphisms in the development of pancreatitis as well as the role of genetic screening for the polymorphism in the general population.

## Introduction

With the annual incidence of acute pancreatitis in the United States ranging from 4.9 to 35 per 100,000 persons, the condition remains a very common and costly healthcare concern [1]. Gallstone disease and alcohol abuse remain the most common etiologies for acute pancreatitis in the United States, accounting for a combined 75% of cases with an identifiable etiology, while alcohol abuse, autoimmune disease, and anatomic variation are considered the most common etiologies for chronic pancreatitis. Unfortunately, 10-40% of patients presenting to their health care provider with acute pancreatitis are labeled as having “idiopathic” pancreatitis, meaning no etiology is identified [2]. Studies have revealed however, that certain genetic polymorphisms may predispose affected individuals to suffer from repeated bouts of pancreatitis throughout their lives. At present, genetic etiologies are thought to be responsible for 8.7% of total pancreatitis cases [3]. We describe the case of a middle-aged Caucasian male with a serine peptidase inhibitor, Kazal type 1 (SPINK-1) polymorphism who presented with his eighth episode of acute pancreatitis within the span of one year [Abstract: Leon D. Averbukh, Marianna G. Mavilia. SPINK-1 Polymorphism as a Pancreatitis Risk Factor. Annual Scientific Meeting of the American College of Gastroenterology; October 2018].

## Case presentation

**Figure 1:**
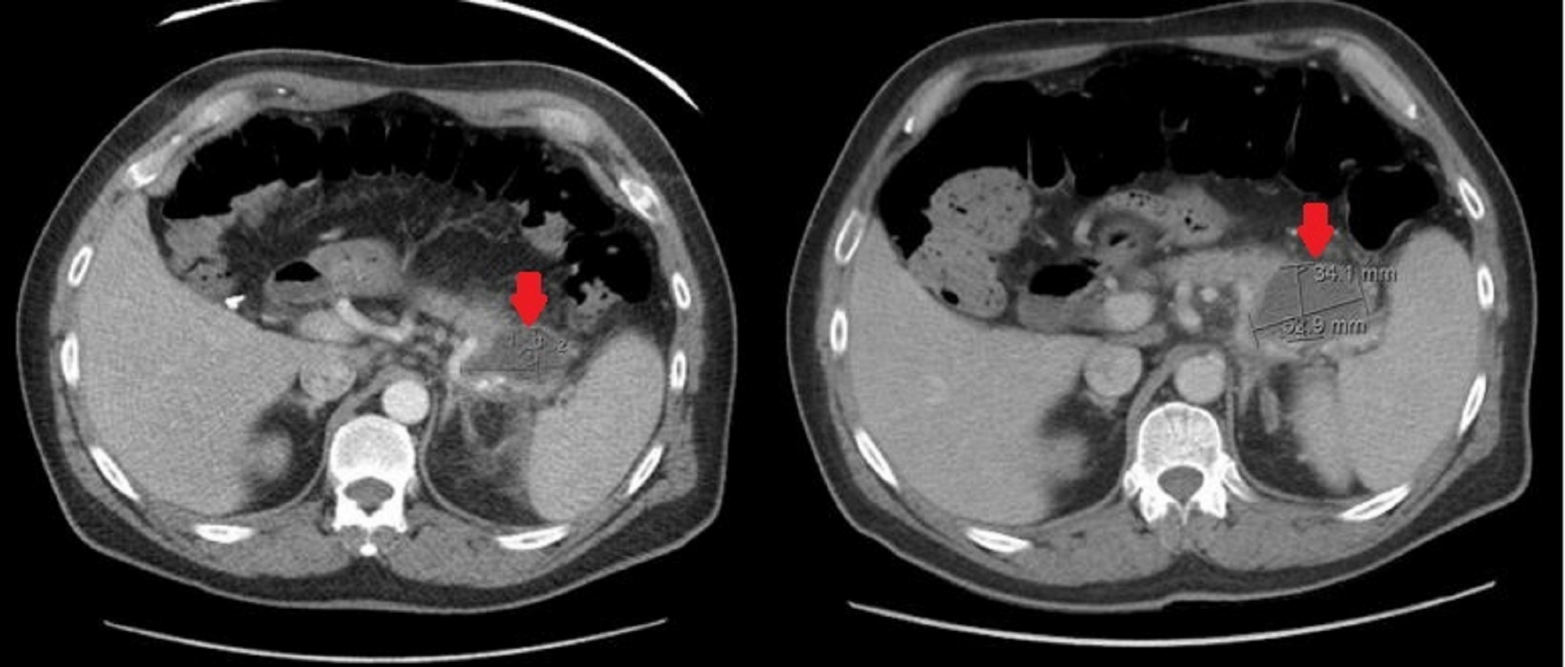
A 5.2 x 2.7 cm cystic lesion was noted on initial computed tomography (CT) abdomen with contrast on prior admission (left, red arrow). Subsequent CT abdomen with contrast two months later (on current admission) showed the lesion had increased to 5.3 x 3.4 cm (right, red arrow).

A 56-year-old male with a past medical history significant for chronic pancreatitis with heterogeneous SPINK-1 mutation (tested for at age 27 due to recurrent episodes of acute pancreatitis) presented with severe left lower quadrant (LLQ) abdominal pain. The patient had already experienced seven episodes of acute pancreatitis within the past year alone, with his last hospitalization for the issue roughly one month prior to current admission. The patient had a remote alcohol and smoking history though he denied any use within the past 20 years, and he recently became a vegan in an attempt to reduce his risk for recurrent acute episodes of pancreatitis. On admission, the patient’s vitals were within normal limits and physical exam was significant only for severe LLQ tenderness and hypoactive bowel sounds. Labs revealed an aspartate aminotransferase of 14 U/L, alanine transferase of 12 U/L, alkaline phosphatase of 63 U/L, lipase 43 U/L, total cholesterol 138 mg/dL, triglycerides of 129 mg/dL, and an international normalized ratio of 1.4. Computed tomography (CT) of the abdomen and pelvis with contrast revealed an enlarging known cystic body in the pancreatic tail measuring 3.4 x 5.3 cm. Peripancreatic inflammatory changes were identified surrounding the distal body of the pancreas and extending along the spleen representing a pseudocyst (Figure 1). The patient experienced resolution of symptoms post supportive management and intravenous (IV) hydration. He was discharged home with planned follow-up imaging of the pseudocyst to monitor its stability.

## Discussion

**Figure 2:**
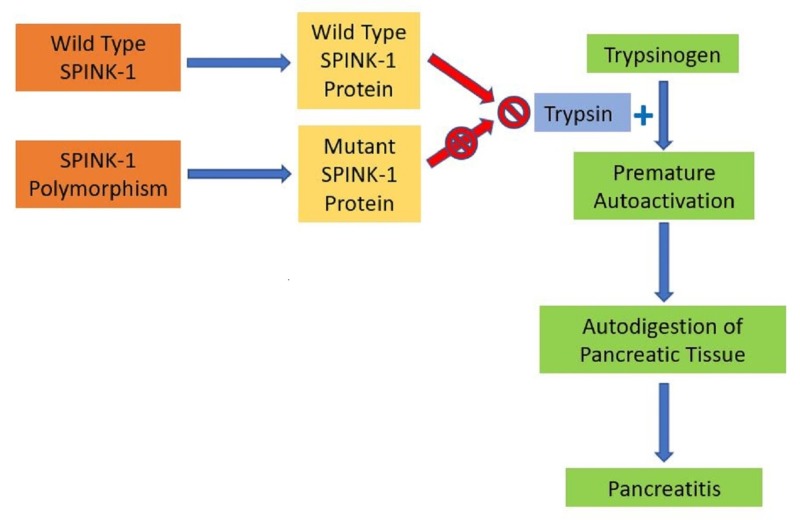
Pathogenesis of the SPINK-1 mutation in the development of pancreatitis. SPINK-1: Serine peptidase inhibitor, Kazal type 1

Currently identified genetic polymorphisms that carry an increased risk for pancreatitis include SPINK-1, cystic fibrosis transmembrane conductance regulator (CFTR), chymotrypsin C alleles (CTRC), and protein coding serine protease 1 (PRSS1). SPINK-1 polymorphisms are rare and seen in only approximately 2% of the US population. The polymorphism has been attributed to 5-6% of all chronic pancreatitis cases and 15-40% of idiopathic pancreatitis cases [4]. The SPINK-1 gene encodes a trypsin inhibitor, which in those with a gene polymorphism, fails to adequately prevent trypsinogen from autoactivating and damaging the pancreas (Figure 2). SPINK-1 mutations predispose patients to acute pancreatitis, earlier onset chronic pancreatitis, and pancreatic cancer [5]. On average, irrespective of gender, those with SPINK-1 polymorphisms who develop pancreatitis do so at a younger age (mid 20’s) when compared to those without it (mid 30’s). Mutations do not follow autosomal dominant or recessive patterns and heterozygotes and homozygotes display similar rates of disease process, making the mutation a disease modifier rather than a direct cause [5]. Treatment for hereditary pancreatitis is the same as that of other etiologies of acute pancreatitis and includes supportive care and abstention from other risk modifiers for pancreatitis such as alcohol and smoking. Rarely, in cases of severe chronic pancreatitis, pancreatectomy with islet cell transplantation is considered [6]. Though regular genetic testing of the general population is neither currently used nor guideline recommended for SPINK-1, early identification of those with polymorphisms may help lead to earlier lifestyle modifications to reduce overall risk of disease development. Our patient’s unfortunate history of pancreatitis is likely secondary to previous insults from alcohol and smoking in the setting of heterozygous SPINK-1 mutation.

## Conclusions

While many of the extrinsic etiologies for acute and chronic pancreatitis are known, genetic components should not be excluded, especially in those presenting with acute episodes at younger ages (20’s vs. 30’s) or recurrent episodes after appropriate lifestyle modifications. Genetic components of pancreatitis are at this time not well understood as there does not appear to be a concrete correlation between polymorphism and the development of pancreatitis. Genetic polymorphisms such as the SPINK-1 mutation serve as a disease risk modifier rather than a directly attributable cause of pancreatitis. While early identification of genetic polymorphisms may help with pancreatitis risk stratification, the as of yet unidentified direct correlation between SPINK-1 mutation and pancreatitis development has led guidelines to recommend against routine childhood screening for the mutation.
